# Genome-wide profiling of the alternative splicing provides insights into development in *Plutella xylostella*

**DOI:** 10.1186/s12864-019-5838-3

**Published:** 2019-06-07

**Authors:** Qian Zhao, Weimin Zhong, Weiyi He, Yiying Li, Yaqing Li, Tianpu Li, Liette Vasseur, Minsheng You

**Affiliations:** 1 State Key Laboratory for Ecological Pest Control of Fujian and Taiwan Crops, Fujian Agriculture and Forestry University, Fuzhou, 350002 China; 2 Institute of Applied ecology, Fujian Agriculture and Forestry University, Fuzhou, 350002 China; 3 College of Life Science, Fujian Agriculture and Forestry University, Fuzhou, 350002 China; 4 College of Plant Protection, Fujian Agriculture and Forestry University, Fuzhou, 350002 China; 5 Department of Biological Sciences, Brock University, 1812 Sir Isaac Brock Way, St. Catharines, ON L2S 3A1 Canada

**Keywords:** IsoSeq, RNA-seq, Diamondback moth (DBM), Alternative splicing, Development, Sex-determination

## Abstract

**Background:**

The diamondback moth (DBM), *Plutella xylostella* (L.), is a major pest of cruciferous crops worldwide. While the species has become a model for genomics, post-transcriptional mechanisms associated with development and sex determination have not been comprehensively studied and the lack of complete structure of mRNA transcripts limits further research.

**Results:**

Here, we combined the methods of single-molecule long-read sequencing technology (IsoSeq) and RNA-seq to re-annotate the published DBM genome and present the genome-wide identification of alternative splicing (AS) associated with development and sex determination of DBM. In total, we identified ~ 13,900 genes (~ 77%) annotated in the DBM genome (version-2), resulting in the correction of 1586 wrongly annotated genes and identification of 78,000 previously unannotated transcripts. We also identified 1804 genes showing alternative splicing (AS) in each of the developmental stages and sexes, suggesting that AS events are ubiquitous in DBM. Comparative analyses showed that these AS events were rarely shared among developmental stages, indicating that they may play key specific roles in regulation of insect development. Further, we found 156 genes showing different AS events and expression patterns between males and females, linking them to potential functions in sex determination*.*

**Conclusion:**

Overall, the *P. xylostella* transcriptome provides the significant information about regulatory alternative splicing events, which are shown to be involved in development and sex determination. Our work presents a solid foundation to better understand the mechanism of post-transcriptional regulation, and offers wider insights into insect development and sex determination.

**Electronic supplementary material:**

The online version of this article (10.1186/s12864-019-5838-3) contains supplementary material, which is available to authorized users.

## Background

Alternative splicing (AS) is the process by which pre-mRNA transcripts can be spliced differentially depending on which exons or portions of exons in a gene are removed from different protein isoforms [1]. The patterns of AS events constantly change under different physiological conditions, allowing organisms to respond to environmental changes through differential genome expressions [1]. For example, herbivory of tobacco hornworm, *Manduca sexta*, feeding on *Nicotiana attenuata* for 5 h reduces AS events by 7.3% when on leaves but increases AS by 8.0% on roots [2].

AS events are associated with development of various organs, such as brain, liver and heart [3]. For instance, *Lef1* exon 6 splicing is regulated in postnatal mouse brain development and T-cell activation [3]. In insects, AS can play an important role in sex determination [4]. In silkworm, doublesex gene (*Bmdsx*) shows a complicated splicing patterns with 10 female-specific, 6 male-specific and 1 splice forms in both male and female [5], which can be used to construct female-specific lethality system [6]. Understanding of alternative splicing may provide important clues in the processes of development and sex differentiation in organisms.

The diamondback moth (DBM), *Plutella xylostella* (L.) (Lepidoptera: Plutellidae), is a global pest of cruciferous vegetables that causes significant damage and economic loss to farmers [7]. A few studies on AS events have focused on the genes with potential functions in resistance against insecticides. The ryanodine receptor (RyR), which is related to resistance development to diamide insecticides in DBM, is comprised of 10 different AS types [8]. Another splicing type in DBM, RyR G4946E variant, confers resistance to diamide insecticides [9]. However, little studies focused on the development or sex differentiation in DBM. We therefore performed the whole-genome wide analysis of AS for further investigation in DBM using the following new technologies.

The single-molecule sequencing technology, known as PacBio (Pacific BioSciences) platform, represents such a novel technique that produces longer reads than the next-generation sequencing (NGS) technologies and has improved the identification of gene isoforms [10, 11]. The single-molecule sequencing method is mainly used to characterize the complexity of transcriptome in plants including maize [8], sorghum [12], strawberry [13], and bamboo [14]. For insects, the PacBio platform has been used to construct the transcriptome map of mitochondrial genome of *Erthesina fullo* [15].

Here, we used the PacBio platform to identify the transcript isoforms with pooled samples of DBM to better understand the development and sex determination of this species. To do so, we used samples from different developmental stages (including eggs, 4 larval instars, pupae and adults) as well as from both sexes. The same pooled samples were also sequenced on the Illumina HiSeq 2000 platform to quantify the gene/isoform expression. Taking the advantage of these two sequencing platforms, we obtained an abundant data set of the DBM transcripts that was far more complex beyond our knowledge. The genome-wide identification of multiple AS events in the DBM genome generated a comprehensive map of post-transcriptional regulation mechanism, which could provide important clues for the future research to elucidate the mechanisms underlying the development and sex-determination in DBM.

## Results

### Characterization of the transcriptome

**Fig. 1:**
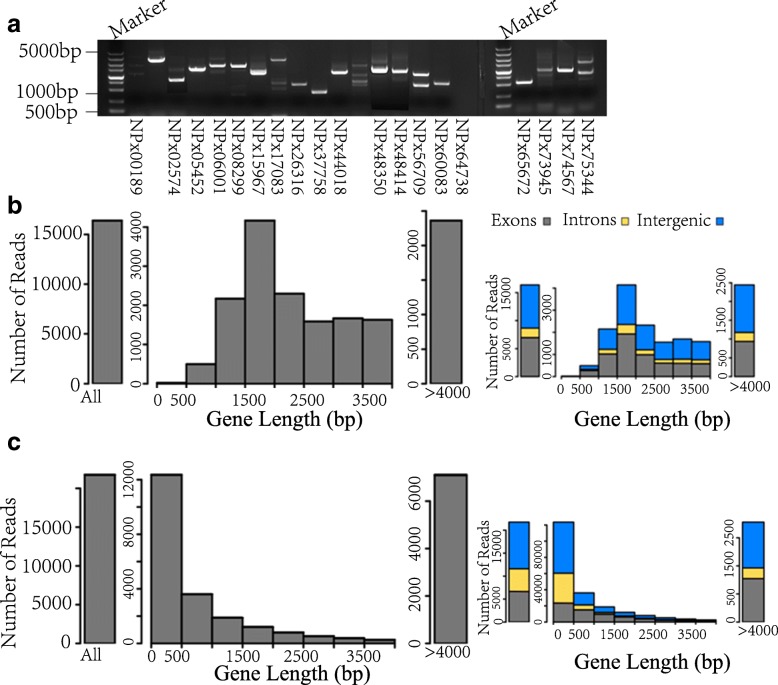
Summary of the sequencing data. **a** Novel transcripts verified by RT-PCR. **b** Basic information of the sequencing data based on Iso-seq. Figure on the left showed read length distributions of Iso-seq; Figure on the right showed read length distributions of Iso-seq among exons, introns and intergenic regions. **c** Basic information of the sequencing data based on RNA-seq. Figure on the left showed read length distributions of RNA-seq. Figure on the right showed read length distributions of RNA-seq among exons, introns and intergenic regions

In order to identify as many transcripts as possible (compared with the Illumina platform [12]), both RNAseq and IsoSeq methods were used to sequence the pooled samples from different developmental stages and sexes. We eventually identified 217,535 (76%) and 16,398 (33%) non-redundant transcripts based on RNA-seq and IsoSeq (Table 1), covering approximately 77% of the gene set. Meanwhile, we also identified 77,648 and 2652 novel transcripts using RNA-seq and IsoSeq methods, respectively (Table 1). We randomly selected 20 candidates to perform RT-PCR experiment, and 19 of them were validated to be true in the DBM genome with a false positive rate of 5% (Fig. 1a). These results further confirmed the reliability of our identification process.

**Table 1:** Data overview of RNA-seq and IsoSeq

Sequencing pattern	Transcripts No.	Non-redundant transcripts No.	Gene coverage (%)	Novel transcripts
RNA-seq	913,851	217,535	76%	77,648
IsoSeq	52,093	16,398	33%	2652

Comparing these two sequencing methods, we found that they covered different gene lengths with most of the IsoSeq reads mapping genes of 1500–2000 bp (Fig. 1b) while most of the RNA-seq reads mapping genes of < 500 bp and the number of genes declining with increasing gene length (Fig. 1c). This might partly result from the short RNA-seq data that were technically difficult to assemble into long transcripts and when preparing libraries for IsoSeq, short fragments (< 500 bp) were removed. As expected, RNA-seq generated higher coverage than IsoSeq and together they further added to the coverage of gene set (Additional file 1: Figure S1A), indicating that merging the two sequencing methods could effectively decrease the error rate.

**Fig. 2:**
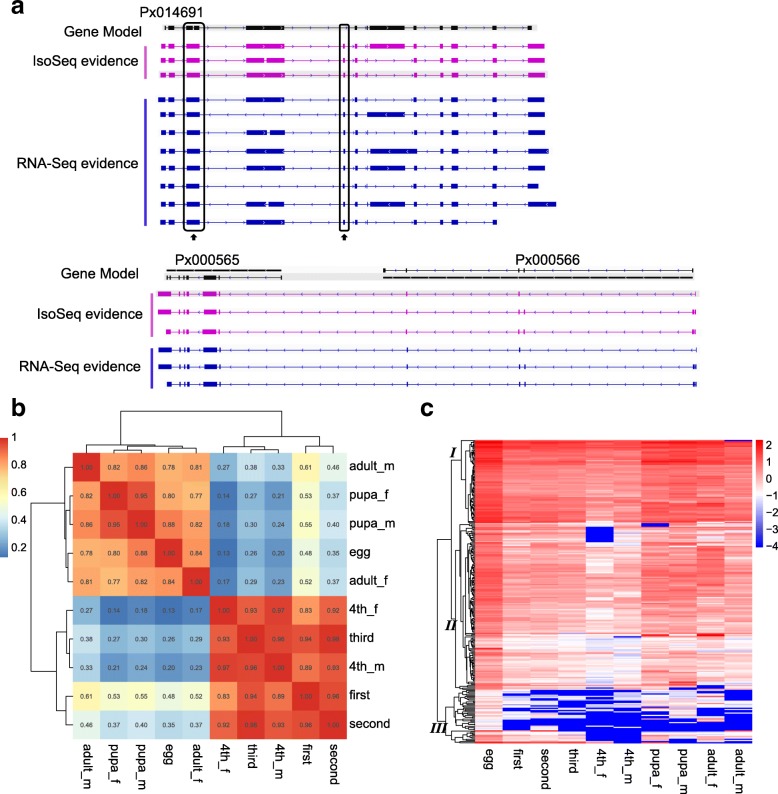
Examples of falsely-annotated genes in the DBM genome. **a** Gene structure of mis-annotated genes*.* The Iso-seq evident was marked by purple and the RNA-seq evident was marked by blue. The box indicated exons and the solid lines represented introns. **b** The distribution of Pearson’s correction coefficients by pairwise misannotated genes. **c** Gene expression pattern analysis of the mis-annotated genes. Heatmap of expression changes along developmental stages was shown by hierarchical clustering. Expression values were calculated as log_10_^FPKM^. Abbreviations: egg, first for first-stage larvae, second for second-stage larvae, third for third-stage larvae, 4th_f for female forth-stage larvae, 4^th^_m for male forth-stage larvae, pupa_f for female pupa, pupa_m for male pupa, adult_f for female adult, and adult_m for male adult

Based on the Isoseq data, we identified some misannotations of genes in the published DBM genome [16]. For example, in the reference genome, Px014691 was mistakenly annotated as two exons while there was a third exon in the gene model (Fig. 2a). We compared the mapped full-length IsoSeq transcripts with the reference genome and found that some adjacent annotated genes overlapped with the single full-length IsoSeq transcript, and were misannotated as two separate genes in the reference genome (Px000565, Fig. 2a). In total, 1586 genes were wrongly annotated as multiple split genes in the DBM reference genome, and could be merged into 699 new loci according to IsoSeq reads (Additional file 6: Table S3). We assumed that these misannotated genes should have shared the same promoters and exhibited similar expression patterns in different developmental stages or tissues. Calculating Pearson’s correlation coefficient (PCC) for each pair of these misannotated genes, we found that there was a strong bias towards positive correlation (Fig. 2b), suggesting that they could have originated from one locus in the genome. There was a peak of correlation coefficient around 0.3, possibly resulting from the PCC analysis performed with all the mis-annotated genes, some of which might have had stage-specific expression patterns. We further analyzed the expression patterns for these genes and confirmed that some of them exhibited varying expression patterns among different developmental stages (Clusters II and III in Fig. 2c), possibly leading to the low correlations.

Besides, we also identified a significant number of novel transcripts (77,648 and 2652 based on RNA-seq and IsoSeq sequencing methods, respectively) and most of them were validated by RT-PCR. Among the novel transcripts, about half of them (43,561) had not been previously annotated. We further compare the novel transcriptomes produced by RNA-seq and IsoSeq. We found that 68,448 novel transcripts from RNA-seq and 335 novel transcripts from IsoSeq did not overlap (Additional file 1: Figure S1B). We analyzed their length distribution and found that the non-overlapped transcripts from the two methods were apart from each other. For example, IsoSeq tended to find longer transcripts compared to the RNA-seq (Additional file 1: Figure S1B).

## Alternative splicing events

**Fig. 3:**
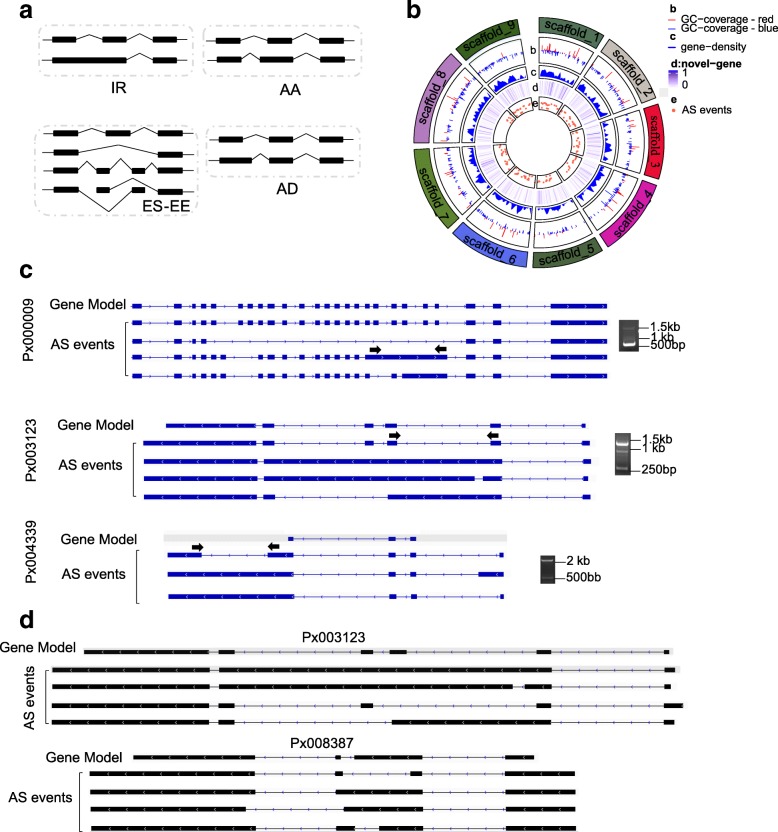
Alternative splicing events in *P. xylostella*. **a** Four types of AS events identified in this study. **b** CIRCOS visualization of data at the genome-wide level. Only 9 scaffolds are shown. From outer to inner circle display GC coverage, gene density, novel genes and AS events respectively. The GC content value higher than the average GC content value was marked by red while lower than average was marked by blue. **c** Examples of AS events in *P. xylostella.* The box indicated exons and the solid lines represented introns. **d** Examples of genes showing different types of AS transcripts. The box indicated exons and the solid lines represented introns

The genome-wide profiling of alternative splicing was performed using the assembled transcriptome by ASTALAVISTA algorithm and identified a total of 1804 genes showing AS events in DBM (Table 2). These AS events were classified into four groups (Fig. 3a), with intron retention (IR), exon skipping (ES-EE), alternative acceptor (AA) and alternative donor (AD) sharing about 4.5, 3.4, 2.7 and 2.7% of the genome, respectively (Table 2, Fig. 3b). We randomly chose 18 events for RT-PCR validation (Fig. 3c; Additional file 5: Table S2) and 16 of them were validated, suggesting that 89% of our identifications were valid.

**Table 2:** Types of alternative splicing events identified with two different sequencing methods

Sequencing method	IR	ES-EE	AA	AD	Total
RNA-seq	378	466	306	317	1151
IsoSeq	496	242	245	224	920
Interaction	66	98	65	53	267
Total					1804

We further analyzed the AS events in each of the developmental stages and sexes to understand their functions. The results showed that AS events existed in every developmental stage as well as in both sexes (Table 3). The number of ES-EE events was relatively similar in every developmental stage (Additional file 2: Figure S2a). Comparative analyses however showed that most types of AS events differed among developmental stages (Additional file 2: Figure S2b). Only 1 ES-EE and 3 AD events were identified as common to all developmental stages. Moreover, multiple splicing modes could operate on the same transcript, leading to several isoforms from a single gene. For example, a lipoprotein receptor in DBM, Px003123, was found to have many AS events resulting in four isoforms (Fig. 3d). Similarly, another X-box binding protein, Px008387, also had different AS events leading to four isoforms (Fig. 3d).

**Table 3:** Alternative splicing events in different developmental stages and sexes where numerical values represent the instar stages, and f for female and m for male

Splicing model	egg	1st	2nd	3rd	4th_f	4th_m	pupa_f	pupa_m	adult_f	adult_m	Total
IR	105	64	69	59	58	61	78	79	50	82	705
ES-EE	74	84	79	71	57	76	89	105	74	104	813
AA	52	48	45	42	31	42	51	61	50	57	479
DD	64	64	53	49	43	58	64	74	65	68	602
Total	259	233	221	201	170	215	254	282	218	277	

### Expression profiles and alternative splicing modes

**Fig. 4:**
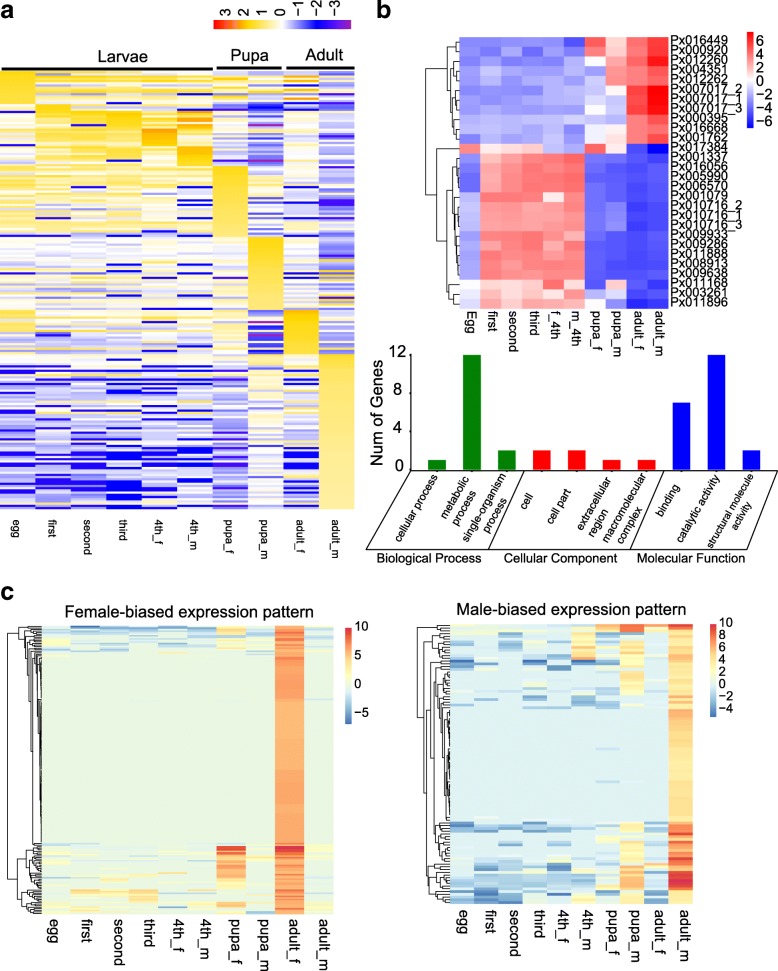
Expression profiling based on RNA-seq. Heatmap showing expression changes among different developmental stages. Expression values were calculated as log_10_^FPKM^. See Fig. 2 for abbreviations of development stages. **a** Genes showing stage-specific expression patterns. **b** Genes showing larval-stage specific expression patterns and their function annotations. **c** Genes showing sex-specific expression patterns. Figure on the left showing female-specific expression. Figure on the right showing male-specific expression

We used RSEM [17] to get the expression value for each gene, and then the edgeR [18] to carry out differential expression profiling. We further defined a stage-specific gene with an AS event as a gene with an expression value 5 times higher than any other stages. With this cutoff, we identified 617 stage-specific expressed genes (t-test, *p* < 0.05; Table 4, Fig. 4a). Most of these genes started to express at the 4th larval stage and peaked in adults, except for the eggs (Table 4). We found that male adults possessed more AS events than female adults (34.7%, Table 4). Some of the profiles at larval stages were undetected (Fig. 4a), which might have resulted from similar expression patterns among some of the isoforms. To verify this, we used WGCNA [19] to perform weighted co-expression profiling and dendrogram analysis. The results showed that larvae and pupae had separate clusters (Additional file 3: Figure S3a and b). The adult male was isolated from all other samples (Additional file 3: Figure S3a and b). Further data analyses showed that 14 genes were highly expressed at the larval stages only, with no difference in expression between males and females. We performed the GO annotation of these genes and found that they were mainly functionally linked to metabolic processes and catalytic activities (*P* < 0.05, t-tests) (Fig. 4b). For example, we identified several GO terms with functions related to serine protease, such as serine-type endopeptidase activity (GO:0004252), serine-type peptidase activity (GO:0008236), and serine hydrolase activity (GO:0017171) (Table 5, Additional file 7: Table S4). We also identified some sex-specific genes, mostly in adults (Fig. 4c).

**Table 4:** Number of proportion of stage-specific expressed genes with AS events with values 5 times higher than any other stages

	egg	1st	2nd	3rd	4th_m	4th_f	pupa_m	pupa_f	adult_m	adult_f	Total
AS events	4	0	0	0	2	1	3	1	8	4	23
Stage-specific genes	81	15	0	6	27	33	68	90	198	105	617
Proportion (%)	4.9	0	0	0	7.4	3	4.4	1.1	4	3.8	3.7

**Table 5:** Go annotation of specific proteins with significantly high expression patterns at larval stages and differentially between males and females

GO ID	Functional description	*P* value	FDR
Proteins with specific expression patterns in larvae stages			
Cellular Component			
GO:0009331	Glycerol-3-phosphate dehydrogenase complex	0.003500	0.041700
GO:1990204	Oxidoreductase complex	0.012500	0.074800
Molecular Function			
GO:0004252	Serine-type endopeptidase activity	0.000005	0.000118
GO:0008236	Serine-type peptidase activity	0.000010	0.000118
GO:0017171	Serine hydrolase activity	0.000010	0.000118
GO:0004175	Endopeptidase activity	0.000058	0.000533
GO:0070011	Peptidase activity, acting on L-amino acid peptides	0.000311	0.002300
Biological Process			
GO:0006508	Proteolysis	0.000540	0.015665
GO:0071704	Organic substance metabolic process	0.001703	0.019009
GO:0008152	Metabolic process	0.001966	0.019009
GO:0005975	Carbohydrate metabolic process	0.003261	0.020091
GO:0046168	Glycerol-3-phosphate catabolic process	0.003464	0.020091
Proteins with different AS events and expression patterns between males and females			
Cellular Component			
GO:0043234	Protein complex	0.005020	0.153015
GO:0031981	Nuclear lumen	0.007547	0.153015
GO:0098796	Transcription elongation factor complex	0.009963	0.153015
GO:0034702	Organelle lumen	0.012343	0.153015
GO:0034703	intracellular organelle lumen	0.012343	0.153015
Molecular Function			
GO:0005524	ATP binding	0.010270	0.116441
GO:0016773	phosphotransferase activity, alcohol group as acceptor	0.000154	0.010854
GO:0005344	Oxygen transporter activity	0.004442	0.100065
GO:0001883	Purine nucleoside binding	0.016417	0.119449
GO:0022890	Inorganic cation transmembrane transporter activity	0.038358	0.165173
Biological Process			
GO:0006464	Cellular protein modification process	0.000382	0.030106
GO:0006812	Cation transports	0.000519	0.030106
GO:0006468	Protein phosphorylation	0.000578	0.030106
GO:0006811	Ion transport	0.010780	0.030106
GO:0042278	Purine nucleoside metabolic process	0.024593	0.220958

Only *p* value < 0.05 are listed. *FDR* False discovery rate. For each of the functions, at most 5 GO terms are listed. The GO terms are all detailed in Additional file 5: Table S4

To verify that alternative splicing might be related to the developmental regulation in DBM, we focused on AS events at specific developmental stages. We identified 23 genes with stage-specific AS events (Additional file 8: Table S5). The types of AS event and the targeted genes differed among developmental stages (Additional file 8: Table S5 and Additional file 9: Table S6). Although ES-EE tended to be the most frequent AS events in DBM, especially in pupae and adults (Table 3), IR was the most prevalent AS events in specific developmental stages (Additional file 8: Table S5). .

We further analyzed the sex specificity in gene expression in DBM, and identified 6902 female related genes and 7609 male related genes. Among them, 346 female-related and 376 male-related genes showed AS events.

### Isoforms produce functional variants

**Fig. 5:**
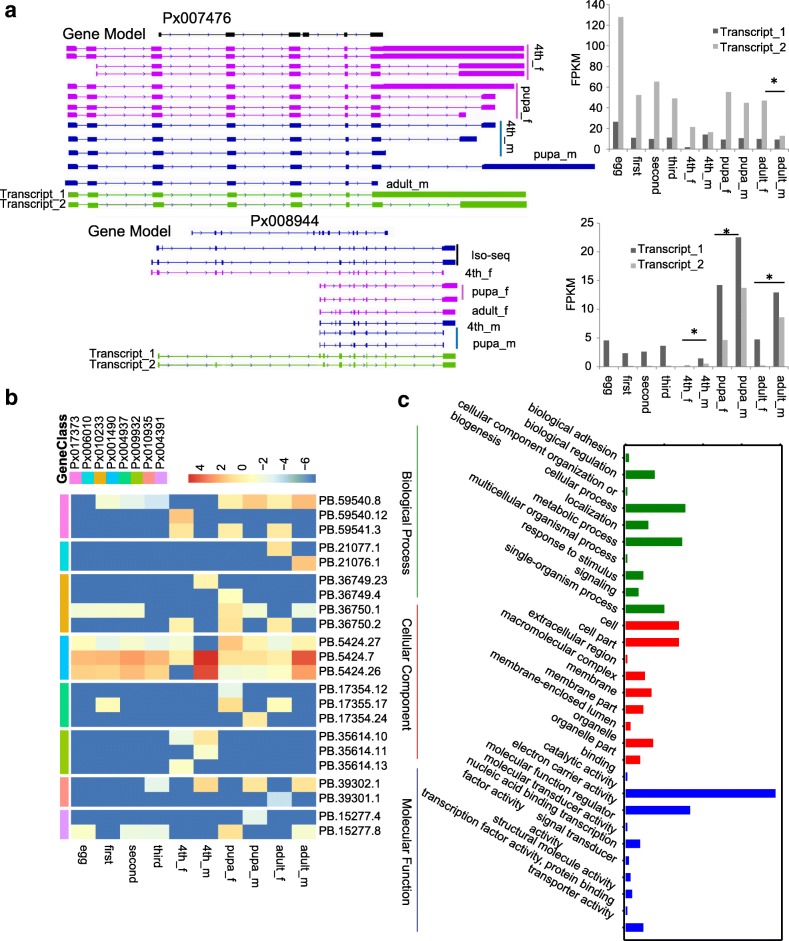
Isoforms produce functional variants. **a** Different alternative splicing transcripts of *tra* and *sxl* in *P. xylostella* and its expression patterns*.* The transcripts of IsoSeq and RNA-seq are merged together to generate the full-length transcripts, which are marked as green. **b** Genes showing sex-specific expression patterns and different alternative splicing in different sexes*.* **c** Functional annotations of these genes

In DBM, the homologous gene of *tra* found in *D. melanogaster* [1] was identified as Px007476. Based on the IsoSeq results, IR was found in this gene (Fig. 5a). Px007476 had multiple transcripts and two of them had high expression values (FPKM > 20) (Fig. 5a). While these two transcripts showed highest expression in eggs, one of them also had an expression level with three times higher in female adults than male adults (Fig. 5a). The *Sxl* locus, Px008944, presented significantly higher expression in later stages than early developmental stages with differential expression between males and females, suggesting different roles in sex determination (Fig. 5a). We also identified the *vis* homolog of *D. melanogaster* in DBM (*e*-value: 7e-25, identity: 82%), which exhibited five different AS events based on IsoSeq results (Additional file 3: Figure S3c).

To illustrate the sex-specific regulation of splicing events, we analyzed different AS events with sex specific expression patterns and found 156 such genes (Fig. 5b, Additional file 10: Table S7). Further functional analysis (GO annotation) was carried out and showed that some GO terms were extremely enriched (*P* < 0.05, Fisher’s exact test, Fig. 5c). For example, we identified several GO terms related to transmembrane movement of ions, ATP binding, and transcription elongation factor complex (Additional file 7: Table S4). We assumed that these genes may be related to reproductive functions and male/female development, which may provide some clues for male/female organ development and sex-determination in DBM.

## Discussion

In this study, we combined the IsoSeq and RNA-seq methods to reconstruct the DBM transcriptome, which covers about 77% of the genome. IsoSeq with full-length non-chimeric reads can significantly improve annotations of the current reference genome. Previous studies in bamboo and maize [14, 20] demonstrate the advantage of single-molecule long read sequencing for investigating poorly assembled loci or novel genes. We re-annotated the transcripts of DBM by IsoSeq and identified 1586 loci that were wrongly annotated as multiple genes in the published reference genome using Illumina sequencing [16].

For the novel transcripts, we speculated that they might have originated from the current experimental design using the samples from multiple developmental stages as compared to the reference genome [16]. The present work was deliberately designed to maximize the entire transcript by collecting samples from each of the development stages and both males and females separately at later stages. Thus, these new transcripts provide useful information for interpreting the development or sex differentiation in DBM. We believe that the gene annotations have been significantly refined based on the IsoSeq sequencing to improve annotations in the reference genome.

### AS identification in DBM

To avoid false positives of AS identification from short assembly of RNA-seq, we set the cutoff of coverage at 0.8, retaining only about ~ 58% of the AS cases for further analyses. For example, before filtration, 3070 identified AS genes were distributed in all developmental stages. Among them, 398 genes showed sex-biased expression patterns. After filtration, 63% of the AS genes were filtered out. Finally, 156 AS cases were identified in our study. By the end, we identified 1804 AS events in DBM, a similar number to other Lepidoptera species, such as silkworm with 1923 AS events [21].

The splicing types were also similar between the two species. In silkworm, about 46% of the identified AS events are intron retaining (IR), which is close to our proportion of IR (44%) in DBM. It is assumed that IR introduces the stop codons to activate non-mediated decay, which thereby regulate the development of organisms [22]. In this study, ES-EE dominated with 51% of the AS events. Although only a few species have been analyzed so far, our results are similar to AS events of maize where ES-EE is also the dominant AS type [20]. Our study is consistent with other AS events as previously reported in both lice head and body with ES-EE over-represented [23]. However, in silkworm, IR is over-represented [21]. Based on a previous study, both IR and ES-EE can change the open reading frames (ORFs), possibly resulting in these functional variants of transcripts [20]. Our results underline the complexity of regulation mechanisms in insects.

### AS events with potential functions in DBM

In this study, we identified AS events in the sodium channel coding gene. Voltage-gated sodium channels are the transmembrane proteins necessary for the generation and propagation of action potentials in excitable cells [24]. The sodium channel proteins in DBM have been well characterized and documented [25, 26]. We identified intron retaining and exon skipping in this gene (Px012020). Previous studies have shown that the AS events within this sodium channel are related to pyrethroid- resistance in the field populations of DBM [26], indicating the complexity of the AS in the post-transcription regulation in the DBM resistance to insecticides.

Fourteen genes showing larval-specific co-expression patterns were identified and according to the GO analysis their functions were related to serine protease, such as serine-type endopeptidase (GO:0004252), serine-type peptidase (GO:0008236) and serine hydrolase (GO:0017171) activities. A previous DBM study has reported that serine protease and its homologs are related to immunity [27].

Genes related to sex determination in *D. melanogaster*, such as Sex lethal (*Sxl*), doublesex (*dsx*) and transformer (*tra*), show different spliced isoforms between males and females [1, 28]. In our study, we identified the homologous gene of *tra* in DBM, Px007476, which showed different AS transcripts between males and females. However, *vis* gene was not testis-specific in DBM (based on RNA-seq analysis from our data and Peng et al. [29], suggesting differences in male development between the two species.

To illustrate the sex-dependent regulation of slicing events, we focused on the AS events showing different expression patterns between males and females and identified 156 genes with sex differentiated AS events. Transgene-based genetic sexing methods are being developed for manipulation of pest and are receiving a great deal of research attention [6]. The female-specific lethality system for genetic sexing of silkworm has been developed based on the *dsx* gene in the genome [6]. In *D. melanogaster*, several genes including *sxl* (sex lethal), *tra* (transformer) and *dsx* (doublesex) are well characterized cascades for sex-determination by alternative splicing [1]. And studies of silkworm showed that *Feminizer* (*Fern*), a precursor of a single W chromosome-derived PIWI-interacting RNA (piRNA), is involved in cleaving the mRNA of the Z-linked *Masculinizer* (*Masc*) gene [30, 31]. Transfection of inhibitor RNA of this piRNA resulted in the production of the male-type splice variant of the *Bmdsx*, indicating that the inhibition of this piRNA function leads to masculinization [30, 31]. *Bmdsx* acts at the downstream end of the sex determination cascade in silkworm based on previous study [32]. However, this gene is not covered by reads generated in this study for some particular samples including male/female adults and male/female 4th larvae, which give us little clue to study this transcript. Interestingly, we identified a GO term named as Transcription elongation factor complex that is enriched for these 156 genes. A previous study in *Caenorhabditis elegans* has shown that a Transcription elongation factor, named as *TCER-1*, is required for loss of the germ cell to increase the lifespan [33]. The information about Transcription elongation factor in DBM was largely unknown until now and our result suggested that they might also be involved in the male/female specific development, reproduction or sex-determination in DBM. Our work also provides insights for transgenic studies and prospects for sustainable control of DBM.

## Conclusions

Here we combined the IsoSeq and RNA-seq to present the genome-wide identification of AS events associated with developmental and sex determination of DBM. Totally, we identified about 13,900 genes, with 1586 wrongly annotated genes were corrected in the present study. Also, we identified 78,000 annotated transcripts. Among these transcripts, 1804 genes were identified to show AS, suggesting that AS events are ubiquitous in DBM. However, these AS events were rarely shared among different stages based on comparative analysis, suggesting that they may play key specific roles in regulation of insect development. Interestingly, we also identified 156 genes showing different AS events and expression patterns between males and females, linking them to potential functions in male/female development or sex determination. Overall, our study provides a foundation to understand the mechanism of post-transcriptional regulation of DBM and offers insights into DBM development and sex determination.

## Methods

### Sample collection

The DBM of Fuzhou-S strain [16] was collected from cabbage (*Brassica oleracea var. capitata*) crop in Fuzhou (26.08°N, 119.28°E) in 2004 and since then, reared on potted radish seedlings (*Raphanus sativus* L.) in rearing cages without exposure to insecticides at 25 °C. Individuals from this colony were collected every 12 h throughout the life cycle from eggs to adults. Starting at the 4th instar, we separately collected males and females as sex could be identified. In total, 60 individuals were collected (*n* = 6 eggs, *n* = 5 1st instar larvae, n = 6 2rd instar larvae, n = 5 3rd instar larvae, n = 6 4rd instar male larvae, n = 6 4rd instar female larvae, *n* = 7 male pupae, n = 7 female pupae, n = 6 male adults, and n = 6 female adults). For each developmental stage, individuals were pooled to form the samples to be sequenced. Collected samples were immediately frozen in liquid N_2_ and stored in freezer at − 80 °C.

### Illumina RNA-Seq library construction and sequencing

Each of the 10 samples (eggs, 1st larvae, 2nd larvae, 3rd larvae, 4th male larvae, 4th female larvae, male pupae, female pupae, male adults, female adults) were grinded in TRIzol regent on the dry ice, and then processed following the protocol of RNAeasy Mini Kit (manufacturer: Qiagen). To remove DNA, total RNA was treated with DNaseI (manufacturer: Qiagen). We used the Agilent 2100 with an RNA Integrity Number (RIN) value to assess the total RNA quality. RNA samples with RIN > 8 were used for following analyses. RNA-seq libraries were constructed using 20 μg total RNA from all samples (2 μg for each sample) following the protocol from ScriptSeq kit with insert size from 280 bp ~ 350 bp [20]. Finally, Illumina HiSeq 2500 platform was used to generate the PE (Paired-end) reads.

### Illumina data analysis

Raw reads with > 2 N bases were first filtered. Remaining reads were processed by clipping adapter, removing low quality bases less than 20. We filtered the short reads (shorter than 16 nt) using the FASTX-Toolkit (Version 0.0.6, http://hannonlab.cshl.edu/fastx_toolkit/commandline.html) (Additional file 4: Table S1). The transcripts of DBM were first obtained by de novo assembly. The reads were assembled using Trinity (−-max_memory 800G –CPU 20 –normalize_reads) [34]. Since the annotated genome [16] (hereafter called reference genome) was available, the transcriptome was reassembled based on the Trinity genome-guided algorithm. These two assembled transcripts were then integrated to generate a comprehensive transcriptome by PASA with default parameters [35].

### PacBio long-read sequencing

Total RNA of the 10 samples were mixed together in equal parts (2 μg for each sample). The total RNA was reverse transcribed using the Clontech SMARTer™ PCR cDNA synthesis Kit with anchored oligo(dT)30 as the primer. The double-strand cDNA was then amplified with LD-PCR (Long-Distance PCR) using Advantage 2 PCR Kit. We used the BluePinppin size selection system (Sage Science, Beverly, MA) to generate cDNA fractions with different sizes including 0.5-1Kb, 1–2 Kb, 2–3 Kb and > 3 Kb. These libraries were then constructed with the Pacific Biosciences’ SMRTbell Template Prep Kit 1.0, following the manufacturer’s protocol. A total of six SMRT cells (2 SMRT cell for libraries of 1–2 Kb; 2 SMRT cell for libraries of >3Kb; one SMRT cell for libraries of 0.5–1 Kb, and; one SMRT cell for libraries of 2–3 Kb) were sequenced on the PacBio RS II platform.

### Analysis of PacBio data

The DBM genome sequences and the annotated gene models were downloaded from DBM database (http://iae.fafu.edu.cn/DBM/). The consensus tool SMRT Analysis 2.3.0 (https://www.pacb.com/documentation/smrt-analysis-software-installation-v2-3-0/) was used to get reads of insert with the following parameters: -n 20 –output = cellE –fofn = input.E.fofn –minFullPasses = 1 --minPredictedAccuracy = 80 --minLength = 100. Then full-length and non-full-length transcripts were classified with the pbtranscript classify script (https://github.com/PacificBiosciences/cDNA_primer/wiki/RS_IsoSeq-(v2.3)-Tutorial-%231.-Getting-full-length-reads). The pbtranscript cluster script was used to isoform-level clustering (ICE). The results were polished with Quiver (https://github.com/PacificBiosciences/cDNA_primer/wiki/RS_IsoSeq-(v2.3)-Tutorial-%232.-Isoform-level-clustering-(ICE-and-Quiver)). We employed gmap tools to map the high quality PacBio transcripts to the genome with the default parameters: -f samse -z sense_force -t 20 -n 0 [36]. Then the samtools was used to sort the sam format with the following parameters [37]: -k 3, 3 -k 4,4n. Finally, the redundant transcripts were removed using collapse_isoforms_by_sam.py (https://github.com/PacificBiosciences/cDNA_primer/wiki/tofu-Tutorial-(optional).-Removing-redundant-transcripts).

### Identification of differential AS events

In this study, we focused on the four main modes of alternative splicing: IR, intron retention; ES-EE, exon skipping, including mutually exclusive exon; AA, alternative 3′-acceptor, and; AD, alternative 5′-donor. We identified the AS events in the 10 samples (i.e. every developmental stage and both sexes) based on the GTF file using ASTALAVISTA algorithm [38]. To avoid false positives in AS identification because of the short assembly results of RNA-seq, we only focused on the assembly transcripts covering 80% of the PacBio transcripts or gene model.

### Expression profiling of different isoforms

In order to get the expression profile, we used RSEM [17] to calculate the expression level for each of the 10 samples. The gene expression levels were measured and normalized as FPKM (fragments per kilobase of transcript, per million fragments sequenced). The transcripts with FPKM < 1 across all the samples were left out from further analysis.

### GO annotation and enrichment analysis

We assessed whether the molecular function, biological process, and pathway terms were over-represented in the target gene set using the OmicShare tools (www.omicshare.com/tools). For each gene set, we computed an expected number of genes for different biological processes based on their curated representation in the reference genome. The statistical significance of the functional GO slim enrichment was evaluated using Fisher’s exact test (*P* < 0.05).

### Statistical analysis and data presentation

The statistical analyses and plots were performed using R package. Heat maps were generated by pheatmap R package. All genome features with RNA-seq and PacBio tracks are visualized by Integrative genomics viewer (IGV) [39].

### RT-PCR validation

RT-PCR was performed to validate the AS events and the existence of the novel transcripts that were not identified prior of this study. Total RNA was prepared according to the same protocols as for Illumina RNA-Seq library construction and sequencing. cDNA was prepared using GoScript Reverse Transcription System (Promega). Primers used to validate the identified AS events and novel transcripts are included in Additional file 5: Table S2. For LightCycler reaction, the master mixture included: 8 μl water, 12.5 μl 2xPhanta Max Buffer (2 mM), 0.5 μl dNTP Mix (10 mM), 1.5 μl cDNA, 1 μl forward primer (0.1 μM), 1 μl reverse primer (0.1 μM) and 0.5 μl Phanta Max Super-Fidelity DNA Polymerase (1 U/μl). The PCR conditions were as follows: 95 °C for 3 min, 34 cycles of 95 °C for 15 s, 50°~ 60 °C (depending on different genes) for 15 s, and 72 °C for 60 s, followed by a final extension period of 72 °C for 5 min.

## Additional files

Additional file 1: **Figure S1. **(**a**) Percentages of coverage of the DBM genome based on different methods of sequencing based on Iso-Seq and RNA-seq and for different components of the genome. (**b**) The length distribution of novel transcripts. The x-axis is transcript length (Log_2_^nt(length)^) and the y-axis density. (TIF 2237 kb)

Additional file 2: **Figure S2.** AS events identified at the DBM genome-level. (**a**) Distribution of different types of alternative splicing events at different developmental stages. Egg, 1st, 2nd, 3rd, 4th_M, 4th_F, Pu_F, Pu_M, Adult_F and Adult_M represent stages of egg, first-stage larvae, second-stage larvae, third-stage larvae, male forth-stage larvae, female forth-stage larvae, male pupa, female pupa, female adult and male adult. (**b**) Distribution of the different AS events among developmental stages including those are common to all stages. (TIF 2122 kb)

Additional file 3: **Figure S3.** Weighted co-expression patterns and the dendrogram analysis of gene expression. (**a**) Weighted co-expression patterns. (**b**) Dendrogram analysis of gene expression. (**c**) Expression patterns analysis of *Px-vis*. The box indicated exons and the solid lines represented introns. (**d**) Expression patterns of different AS isoforms for this gene among different developmental stages was also shown. (TIF 2234 kb)

Additional file 4: **Table S1.** Reads info of RNA-seq. (DOCX 17 kb)

Additional file 5: **Table S2.** Primers used for the RT-PCR validation. (DOCX 20 kb)

Additional file 6: **Table S3.** Mis-annotated genes list identified by Iso-Seq. (TXT 199 kb)

Additional file 7: **Table S4.** GO annotations for the 156 genes. Only the terms showing *p* < 0.05 were listed. (XLSX 18 kb)

Additional file 8: **Table S5.** Developmental stages specific-expressed genes showing alternative splicing. (DOCX 20 kb)

Additional file 9: **Table S6.** Gene showing AS event during developmental stages and sexes. (XLS 62 kb)

Additional file 10: **Table S7.** Genes showing isoforms produce functional variants (XLSX 18 kb)

## Data Availability

All data are included in this article and its supplementary information. The RAW data is available at http://bigd.big.ac.cn with project ID: PRJCA001194.

## Abbreviations

AA: Alternative acceptor
AD: Alternative donor
AS: Alternative splicing
DBM: Diamondback moth
ES-EE: Exon skipping
IR: Intron retention
IsoSeq: Single-molecule long-read sequencing techmology
PCC: Pearson’s correlation coefficient
