# Supplementary figures and images for: Genome-wide profiling of the alternative splicing provides insights into development in Plutella xylostella
Source: BMC Genomics. 2019 Jun 7;20:463. doi: 10.1186/s12864-019-5838-3 (PMC6556048; doi:10.1186/s12864-019-5838-3)

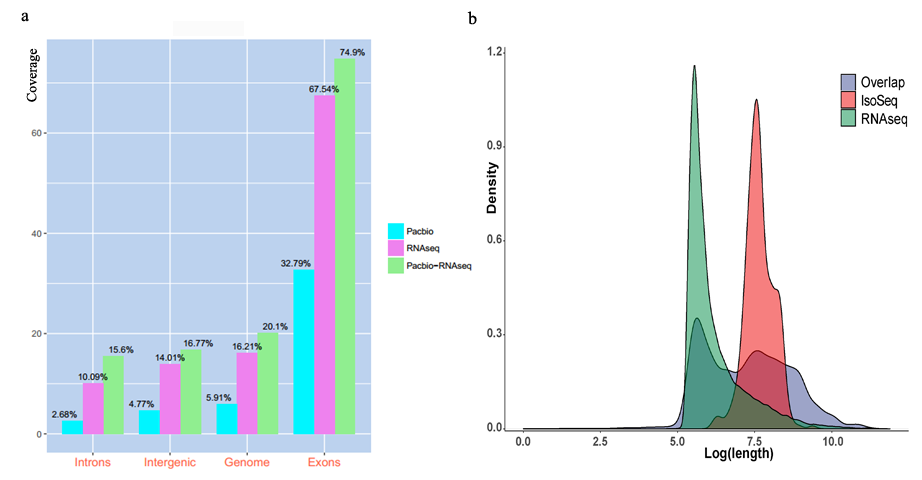

Supplement: Supplementary file 1 — Figure S1. (a) Percentages of coverage of the DBM genome based on different methods of sequencing based on Iso-Seq and RNA-seq and for different components of the genome. (b) The length distribution of novel transcripts. The x-axis is transcript length (Log2nt(length)) and the y-axis density. (TIF 2237 kb) [file 12864_2019_5838_MOESM1_ESM.tif]

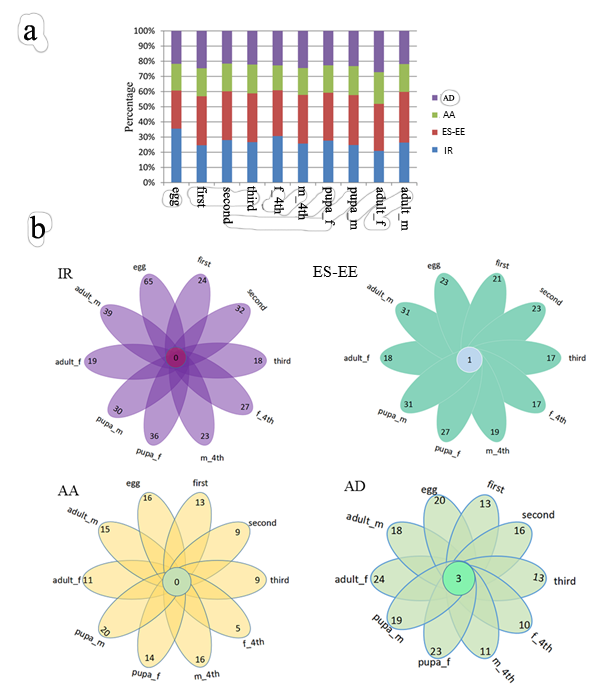

Supplement: Supplementary file 2 — Figure S2. AS events identified at the DBM genome-level. (a) Distribution of different types of alternative splicing events at different developmental stages. Egg, 1st, 2nd, 3rd, 4th_M, 4th_F, Pu_F, Pu_M, Adult_F and Adult_M represent stages of egg, first-stage larvae, second-stage larvae, third-stage larvae, male forth-stage larvae, female forth-stage larvae, male pupa, female pupa, female adult and male adult. (b) Distribution of the different AS events among developmental stages including those are common to all stages. (TIF 2122 kb) [file 12864_2019_5838_MOESM2_ESM.tif]

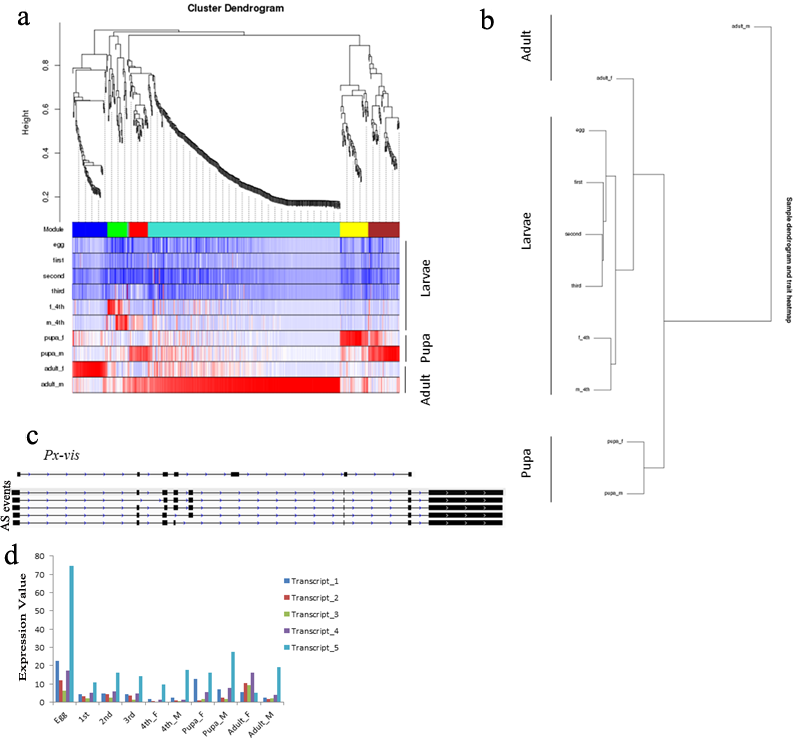

Supplement: Supplementary file 3 — Figure S3. Weighted co-expression patterns and the dendrogram analysis of gene expression. (a) Weighted co-expression patterns. (b) Dendrogram analysis of gene expression. (c) Expression patterns analysis of Px-vis. The box indicated exons and the solid lines represented introns. (d) Expression patterns of different AS isoforms for this gene among different developmental stages was also shown. (TIF 2234 kb) [file 12864_2019_5838_MOESM3_ESM.tif]
