# Supplementary material for: Genome-wide profiling of the alternative splicing provides insights into development in Plutella xylostella
Source: BMC Genomics. 2019 Jun 7;20:463. doi: 10.1186/s12864-019-5838-3 (PMC6556048; doi:10.1186/s12864-019-5838-3)
Supplement: Supplementary file 4 — Table S1. Reads info of RNA-seq. (DOCX 17 kb) [file 12864_2019_5838_MOESM4_ESM.docx]

Additional file 4: Table S1: Reads information of RNA-seq.

| Sample | Length | Raw reads | | Clean Reads | |
| --- | --- | --- | --- | --- | --- |
|  |  | Read Number | Base Number | Read Number | Base Number |
| Egg | 150 | 89,552,334 | 13,432,850,100 | 59,940,954 | 8,991,143,100 |
| First stage larvae | 150 | 91,312,102 | 13,696,815,300 | 58,396,286 | 8,759,442,900 |
| Second stage larvae | 150 | 68,695,266 | 10,304,289,900 | 59,614,984 | 9,942,247,600 |
| Third stage larvae | 150 | 80,888,860 | 12,133,329,000 | 58,883,308 | 8,832,496,200 |
| Female forth stage larvae | 150 | 82,952,664 | 12,442,899,600 | 58,673,278 | 8,800,991,700 |
| Male forth stage larvae | 150 | 93,162,964 | 13,974,444,600 | 58,247,514 | 8,737,127,100 |
| Female pupa | 150 | 90,763,986 | 13,614,597,900 | 59,070,662 | 8,860,599,300 |
| Male pupa | 150 | 80,366,042 | 12,054,906,300 | 58,188,202 | 8,728,230,300 |
| Female adult | 150 | 72,811,924 | 10,921,788,600 | 58,372,240 | 8,755,836,000 |
| Male adult | 150 | 81,049,876 | 12,157,481,400 | 58,798,886 | 8,819,832,900 |
