# Supplementary material for: Genome-wide profiling of the alternative splicing provides insights into development in Plutella xylostella
Source: BMC Genomics. 2019 Jun 7;20:463. doi: 10.1186/s12864-019-5838-3 (PMC6556048; doi:10.1186/s12864-019-5838-3)
Supplement: Supplementary file 5 — Table S2. Primers used for the RT-PCR validation. (DOCX 20 kb) [file 12864_2019_5838_MOESM5_ESM.docx]

Additional file 5: Table S2 Primer lists used in RT-PCR.

| **Gene ID** | **F** | | **R** | **Validation** |
| --- | --- | --- | --- | --- |
| AS Events |  | |  |  |
| Px001490_1 | AACAGCGATAGAGATT | | AAATCCTTGAATCCAT | + |
| Px001490_2 | AACAGCGATAGAGATT | | TTGGACAGACCAGTAT |  |
| Px007522_1 | TCATCCCAATAGTCCTG | | AAAATCCTATCCTAACC | - |
| Px007522_2 | ACTCCAACATCTAATCC | | TTTTGACCCAACTTTTC |  |
| Px010483_1 | TGTATGTGATTTTTCTG | | TTCATCCTATCTGTAAC | + |
| Px010483_2 | CACGATTTTATTACACT | | TGTTAGTTTGTGGGGTC |  |
| Px002795_1 | AAGAACGCTCAGAACAT | | GCAAGTAACAATCAATC | + |
| Px002795_2 | AAAAAATGTCAACGAAAC | | GCATTGAAAGCATAATTC |  |
| Px001712_1 | TATCTCAGAAGCCAACC | | TCACTCTTGTAGAACCC | + |
| Px001712_2 | GAACGCAGAAGAAAACG | | TCATTCTCACTAACATT |  |
| Px007030_1 | CTTTCCATCTCCTTTAT | | ATGTAGCCCTTGAAAAT | + |
| Px007030_2 | GCGTGACCCCGTATTCT | | AACCTCAACGAAGTCAG |  |
| Px012488_1 | AAAGACAAAAGCAGCAG | | TGAGGGCACATTCCAGG | + |
| Px012488_2 | GTTTGTTGACACTTCTG | | CAGAAAGTAGAAAAGGT |  |
| Px011663_1 | ATCATTGTTGGACATAGC | | TTGTGAGGTGGTCAAGAG | + |
| Px011663_2 | TTGATAATAAAATCTGAG | | AGTTTTTGATTTGTGAGG |  |
| Px000969_1 | CCAAATGGTGTTAGACG | | ACTTGAGCGAGGTGTAG | + |
| Px000969_2 | TCCAATGAAATAAAGTT | | TTATTGAAGTCCTGATT |  |
| Px003123_1 | ACCTCCATACCATCATC | | GCTTGTCACAGTTGGTC | + |
| Px003123_2 | GCCCAAGTGCCAGTATTC | | CGTCAACGGCTGTAGAAG |  |
| Px003123_3 | TTAGCCTTCCGAACCTC | | GCAAGCATTTTCATCGG |  |
| Px001937_1 | CAGGAAACAGCGAAAATG | | GATGTTTGCTCAGATGCG | + |
| Px001937_2 | CTATTTACTACTCACGC | | GGGGTAGGAGATTGAGG |  |
| Px012488_1 | AAAGACAAAAGCAGCAG | | TGAGGGCACATTCCAGG | + |
| Px012488_2 | GTTTGTTGACACTTCTG | | CAGAAAGTAGAAAAGGT |  |
| Px016332_1 | GATGTCCAGGTCAGGGG | | GTGCCATCTACAACAAC | + |
| Px016332_2 | GCTTTCGTGGAACCTTT | | CAGATAGCCTCAGCGGT |  |
| Px000009_1 | ATTTGTCTCTCAAGGTAG | | GTTACCTTTGGCATAGTC | + |
| Px000009_2 | ATTGCTGTATCTGTTAT | | TTTGATTCTATGATGGT |  |
| Px000009_3 | CTTCATTAGTCGGGTCT | | ATCATCGGCTGTCTTGT |  |
| Px004339_1 | ATTACCCCCTTACCCCT | | CTTGCCGGACATCTTTT | + |
| Px004339_2 | GTATACACCAGTGCAAC | | TTACTTGGGTTCCGTTT |  |
| Px013988_1 | TCGTGGAGAGAGCCCTT | | GACTGGATGCCCTGGTT | + |
| Px013988_2 | CTAACTGAAAAGCAAAG | | TTACTGAGGATTCTTAG |  |
| Px006969_1 | CGTCAACGGCTGTAGAAG | | ATTACCCCCTTACCCCT | + |
| Px006969_2 | CTTGCCGGACATCTTTT | | GTATACACCAGTGCAAC |  |
| Px002303_1 | TAGACGGAACAAAAGTAC | | TGAAAATGAAGACAAATG | - |
| Px002303_2 | AGAGCCAGTTCAACGATG | | TCAAGAACTACCAAAACC |  |
| Novel transcripts |  | |  |  |
| NPx00189\|scaffold_1:709946-718751(-) 1626 | ATCAGAAGTGAAAGTGG | | ACTCTTGTGAGGGAAAT | - |
| NPx02574\|scaffold_104:593117-596037(+) 2921 | | GATTATGAAACCGCAACC | GATTTTACGCTCTGTGAC | + |
| NPx05452\|scaffold_1115:20078-21440(+) 1012 | | TAACATTTAGGCTTATCGT | AAACTATTCAGGACTTATG | + |
| NPx06001\|scaffold_113:1039472-1042086(-) 2029 | | AAAGAGCAAGTCAGGTC | ACAACATAGTTCCCTTT | + |
| NPx08299\|scaffold_120:346162-355393(+) 2293 | | ATGCTTTTAGGCTTTAC | CTCATCTTCGCTTACTC | + |
| NPx15967\|scaffold_149:598155-600351(+) 1473 | | AGTGTAGCGACATCAGG | ACAAACCAGTTCTCAGT | + |
| NPx17083\|scaffold_153:267620-269390(+) 1331 | | CCATCCATCCGTAGAGC | GGCTATGCTTGGCTTTC | + |
| NPx26316\|scaffold_199:831047-833702(-) 2121 | | GCACCAACGAAAATAGC | AGCACCAGCGTGTTCCT | + |
| NPx37758\|scaffold_275:515487-516304(-) 767 | | ATTTTAGAACACAGCGT | AAGGTGGTTTTATTCTG | + |
| NPx37974\|scaffold_277:147874-148388(+) 515 | | TCAGTTTTGGCAGTTCT | TTTCAATAACTGGCAAC | + |
| NPx44018\|scaffold_319:239984-246027(+) 1367 | | ACAATGGACCAAGGAAAG | TTAGTTCAGGCAGTTTCT | + |
| NPx48350\|scaffold_363:84236-85433(+) 938 | | GCAACACCCTGTATTAG | CTGTGATTGGTGGAACG | + |
| NPx48414\|scaffold_364:104509-106468(+) 1495 | | AACAGATGTAAATGGCG | CAGACCAGGACAAAGTG | + |
| NPx56709\|scaffold_464:148307-149766(+) 1432 | | TCCTTTAGACATCCATT | CTACCTGTCAAGAAAAC | + |
| NPx60083\|scaffold_509:100247-101479(-) 735 | | TAGCGACCAGTGGCAAC | AACCAGAGCCTTACACG | + |
| NPx64738\|scaffold_59:1361718-1362534(+) 778 | | GTGAGAGTGAACTAAAT | TTTACAACAGTTTTCTC | + |
| NPx65672\|scaffold_61:432390-433172(+) 784 | | GTAAGTAATCGTTTTTGT | CGGTTTTGAATCCAGTTT | + |
| NPx73945\|scaffold_83:67702-71674(-) 1130 | | TCATTGGAAGGAAACCCG | AATACCTGTTGCTTGGAT | + |
| NPx74567\|scaffold_86:36544-44937(+) 1096 | | ACTGAAAATAGTGCTGG | AGTCAGTTATGAATGGC | + |
| NPx75344\|scaffold_89:721391-722325(+) 916 | | TATTGCGATGTTGGAGG | TTGTGTTGTTTTGGCAT | + |

Transcripts were verified by PCR were marked with “+”.
