# Supplementary material for: Genome-wide profiling of the alternative splicing provides insights into development in Plutella xylostella
Source: BMC Genomics. 2019 Jun 7;20:463. doi: 10.1186/s12864-019-5838-3 (PMC6556048; doi:10.1186/s12864-019-5838-3)
Supplement: Supplementary file 8 — Table S5. Developmental stages specific-expressed genes showing alternative splicing. (DOCX 20 kb) [file 12864_2019_5838_MOESM8_ESM.docx]

Additional file 8: Table S5. Developmental stage-specific genes showing alternative splicing events based on RNA-seq and IsoSeq data.

| **gene_ID** | **Stages** | **AS_type** |
| --- | --- | --- |
| Px003282 | egg | AD |
| Px009380 | egg | AA |
| Px010864 | egg | AD |
| Px010840 | egg | AA,ES |
| Px009290 | f_4th | IR |
| Px005995 | f_4th | ES |
| Px015683 | m_4th | IR |
| Px003682 | pupa_m | IR |
| Px008690 | pupa_m | ES |
| Px010098 | pupa_m | AD,IR |
| Px013417 | pupa_f | ES |
| Px001293 | adult_f | IR |
| Px012663 | adult_f | AA |
| Px003143 | adult_f | IR |
| Px006362 | adult_f | IR |
| Px011926 | adult_m | AD,ES |
| Px001584 | adult_m | IR |
| Px007931 | adult_m | ES |
| Px004441 | adult_m | IR |
| Px009403 | adult_m | AD |
| Px011817 | adult_m | AA |
| Px003114 | adult_m | AA |
| Px004540 | adult_m | ES |
